# Thinking Outside the Box in Mediastinal Lymphoma Management

**DOI:** 10.1007/s44228-023-00045-7

**Published:** 2023-05-17

**Authors:** Marwa Alhamss, Riad El Fakih

**Affiliations:** 1 College of Medicine, Alfaisal University, Riyadh, Saudi Arabia; 2 Section of Adult Hematology/HSCT, Oncology Centre, King Faisal Specialist Hospital and Research Centre, PO Box 3354, Riyadh, 11471 Saudi Arabia

**Keywords:** Mediastinal Gray zone lymphoma, Classical Hodgkin lymphoma, Primary mediastinal B-cell lymphoma

To the Editor,

Mediastinal lymphomas can be challenging to manage for several reasons, including the acuity of presentation and the difficulties to secure a biopsy for appropriate classification of the disease and management thereafter. The mediastinum is a narrow area where vital structures are located or pass-through. Frequently, physicians are faced with tumors restricted to that area. Patients typically have nonspecific manifestations resulting from compression of surrounding structures. Often, patients are sick and quick tissue diagnosis is needed to start therapy. However, the area is not easily accessible, and sampling is frequently suboptimal which makes the establishment of an accurate diagnosis challenging. Lymphomas are the most common primary malignancy involving the mediastinum [1]. Biopsy is a prerequisite to accurately classify lymphomas and subsequently assign patients to the appropriate therapy based on the appropriate classification.

A healthy 17-year-old girl presented with 4-weeks of shoulder pain, cough, and shortness of breath. Chest-x-ray showed mediastinal mass, which was later confirmed by PET/CT-scan (Fig. 1A). The patient then underwent mediastinoscopy and a core biopsy, which showed CD20/CD30-positive large-B-cell lymphoma. Further characterization was not possible due to the limited number of malignant cells in the specimen. A larger biopsy was not attempted, as the patient was clinically deteriorating. She was started on a DA-REPOCH protocol (dose-adjusted rituximab, etoposide, prednisone, vincristine, cyclophosphamide and hydroxydaunorubicin), along with prophylactic intrathecal methotrexate with each cycle of chemotherapy. Interim and end-of-therapy imaging showed complete-remission (CR) (Fig. 1B). Unfortunately, 4 months after finishing therapy she presented with headache and unsteady gait. Brain-MRI showed a dural-based left temporal tumor and a vermis tumor with surrounding edema and mass effect. PET-scan showed hypermetabolic activity at the site of the initial mediastinal mass and two FDG avid CNS lesions (Fig. 1C and D). Securing a biopsy was discussed, but the multidisciplinary tumor board decision was to hold off, because the biopsy from the mediastinum was risky and with an expected low yield, and the biopsy from the brain lesions carried a high surgical risk, which was rejected by the patient and her family. She was then started on a high-dose methotrexate, cytarabine and thiotepa combination, aiming to achieve both systemic and CNS-remission, to be followed by autologous bone marrow transplant (auto-BMT) consolidation. After two cycles of this combination therapy, the patient developed prolonged cytopenia and re-imaging showed stable-disease in the brain as well as the mediastinum. With the cytopenia prohibiting further use of chemotherapy, a decision was made to treat with pembrolizumab, a checkpoint inhibitor. After the first cycle, the patient developed significant clinical improvement, and re-imaging after 3 months of this therapy showed CNS and systemic CR (Fig. 1E and F). She then underwent auto-BMT using a cyclophosphamide and total-body radiation myeloablative preparative regimen, and is still in CR, 24 months post-transplant.

**Fig. 1:**
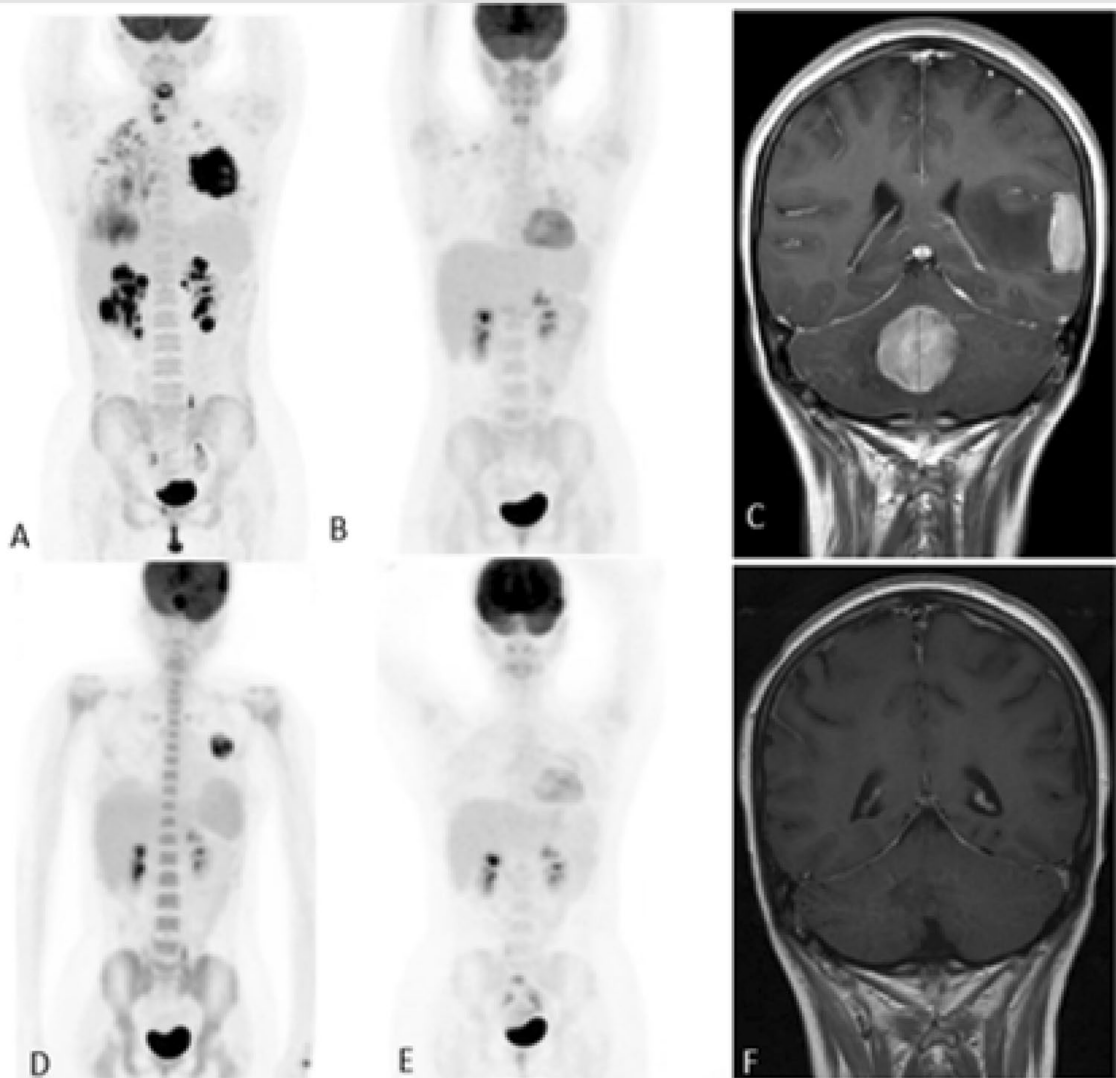
**A** Lymphoma involving the mediastinum, liver, with possible involvement of the kidney, and lung parenchyma. **B** End of therapy imaging showing complete resolution of the previously involved areas. **C** Left temporal dural based tumor and vermian tumor along the fourth ventricular margin, with surrounding vasogenic edema and resultant mass effect. **D** New hypermetabolic activity seen at the site of the initial mediastinal mass. Two new FDG avid CNS lesions. **E** Pretransplant PET scan showing complete resolution of the previously involved areas. **F** Pretransplant brain MRI showing complete resolution of the previously involved areas

Mediastinal lymphomas are aggressive tumors. The classification of lymphomas in general is based on clinical, morphologic, immunophenotypic and molecular characteristics. Most lymphomas can be accurately classified, but some have overlapping features, which makes accurate classification difficult. Classic Hodgkin lymphoma (CHL), primary mediastinal B-cell lymphoma (PMBL) and mediastinal gray-zone lymphoma (MGZL) arise from the thymic B-cells, and are the most commonly encountered mediastinal-lymphomas. Clinically, these are not distinguishable, as they all present with compressive symptoms and predominantly affect young female patients (except for MGZL, which affects males more than females) [1]. Histologically, the presence of Reed-Sternberg cells can help identifying CHL cases but, otherwise, all three entities can have significant similarities. Immunohistochemical staining can be helpful to differentiate these cases, but not uniformly [1]. Table 1 summarizes some helpful differentiating features of these lymphomas [1–7].

**Table 1:** Differentiating features between mediastinal lymphomas

	PMBL	MGZL	NSCHL
Clinical	• Bulky, large mediastinal mass • More common in females • Median age 35[1]	• Bulky, large mediastinal mass • More common in males • Median age 30[2]	• Large bulky mass less common as compared to PMBL and MGZL • More common in females • Median age 30[3]
Morphology	• Sheets of diffuse large lymphocytes with large nuclei [4]	• A mix of features from PMBL and NSCHL [5] • Cells tend to be more various than NSCHL and more pleomorphic than PMBL[1]	• Nodular growth pattern • Broad bands of fibrosis surrounding nodules of lymphoid tissue[6]
Immunohis tochemistry	• CD23 + , weakly CD30 + , and CD15-[4]	• CD45 + , CD30 + , and most often CD15 + [2]	• CD30 + and CD15 + , negative for CD20 [1, 6]
Molecular	• NFkB activation • JAK-STAT pathway activation [7] • Immune evasion, by down regulation of MHC class I and II and upregulation of programmed death ligands[7]	• Copy number gains REL and JAK2[2] • Rearrangement CIITA locus [2] • Gain 8q24(MYC) [2]	• NFkB activation • JAK-STAT pathway activation [7] • Immune evasion, by down regulation of MHC class I and II and upregulation of programmed death ligands[7] • Loss of B2M + [2]

*MGZL* Mediastinal Gray zone lymphoma, *NFkB* Nuclear factor kappa B, *NSCHL* Nodular Sclerosis Classical Hodgkin Lymphoma, *PMBL* Primary mediastinal B-cell lymphoma

We herein report a case that is not uncommon in clinical practice, where a patient presents with symptoms that require quick intervention, while the location of the tumor is difficult to reach and securing a representative biopsy is challenging. Our patient had CD20/CD30-positive tumor. CHL, PMBL and MGZL can have this phenotype. While CD20 can be positive in CHL, this is usually rare; additionally the acute presentation in this patient is atypical of CHL, where the disease progresses at a slower pace [1, 6]. The patient had a mediastinal mass and other extranodal involvement, which is atypical of PMBL, where the disease is usually restricted to the mediastinum upon presentation [1]. In practice, it was difficult to differentiate whether this was PMBL or MGZL, as the patient had overlapping findings, and the specimen collected was suboptimal [8]. The patient received a treatment that is active against both PMBL and MGZL (DA-REPOCH) but relapsed quickly after initial-response. When the relapse involves the CNS, the choice of therapy becomes more challenging, as the number of agents that cross the blood brain barrier is limited [9]. The patient received an active combination of drugs that do cross this barrier (high-dose methotrexate, cytarabine and thiotepa) but, unfortunately, did not respond. The choice of a checkpoint-inhibitor in this patient was based on the shared biological features in mediastinal lymphomas and the published literature backing-up the use of these inhibitors for both systemic and CNS disease involvement [10, 11]. Additionally, this patient failed two lines of very active antineoplastic agents, which guided the treating team to consider agents with different mechanisms of action, rather than cytotoxic agents.

Despite the tremendous advances in lymphoma management, some cases still fall into a grey-zone. Ours illustrates the importance of integrating the biological characteristics of the tumors with the response to prior therapy in order to plan the next line of treatment. It also highlights the importance and the need to divert from the standard conditioning and transplant platform for such cases. Multidisciplinary discussion and shared decision-making by patients and doctors in the context of the available therapies help to navigate the options and choose the appropriate strategy for a successful outcome.

## Data Availability

The data that support the findings of this paper are available from the corresponding author, [Riad El Fakih], upon request.
